# Exploring the experience of meaning-centered group psychotherapy among Chinese cancer patients during active treatment: a descriptive qualitative study

**DOI:** 10.3389/fpsyt.2023.1264257

**Published:** 2023-10-06

**Authors:** Shuman Wang, Mimi Zheng, Yu Zhu, Lijuan Zhang, Xiaoru Li, Hongwei Wan

**Affiliations:** ^1^Department of Nursing, Shanghai Proton and Heavy Ion Center, Fudan University Cancer Hospital, Shanghai, China; ^2^Shanghai Key Laboratory of Radiation Oncology, Shanghai Proton Heavy Ion Hospital, Shanghai, China; ^3^Shanghai Engineering Research Center of Proton and Heavy Ion Radiation Therapy, Shanghai Proton Heavy Ion Hospital, Shanghai, China

**Keywords:** cancer, meaning-centered group psychotherapy, meaning of life, thematic analysis, qualitative research

## Abstract

**Objective:**

Meaninglessness poses a significant psychological challenge for cancer patients, negatively affecting their quality of life and increasing the risk of suicide. Meaning-Centered Group Therapy (MCGP) is an intervention designed specifically to enhance the meaning of life of cancer patients. Extensive research has documented its effectiveness across various cultures and populations. However, limited research has been conducted on the subjective experiences and perspectives of participants engaged in MCGP. Thus, the purpose of this study was to employ a qualitative design to explore the experiences and viewpoints of Chinese cancer patients who have undergone MCGP.

**Methods:**

Within a two-week timeframe following the conclusion of MCGP, semi-structured interviews were administered to twenty-one participants who had engaged in the therapy. The interview data were transcribed and subjected to thematic analysis.

**Results:**

Four main themes were identified: (a) Self-perceived personal change, (b) Overall experience of group therapy, (c) Barriers to participation of MCGP, and (d) Suggestions for future interventions.

**Conclusion:**

Despite the barriers to participation in the MCGP process, the overall experience for Chinese cancer patients undergoing active treatment is valuable and positive, providing multiple benefits. Future studies could explore the adaptation of MCGP to a broader range of cancer populations and diverse study populations.

## 1. Introduction

As the world’s most populous country, China bears the highest burden of new cancer cases and fatalities globally (1). The diagnosis and treatment of cancer inflict a range of adverse psychological effects on patients, including diminished meaning of life, psychological distress, despair, and contemplation of suicide, significantly impacting their quality of life (2, 3). The quest for the meaning of life ranks among the foremost existential concerns within the context of the cancer experience (4–7). A high level of meaning of life predicts better coping with cancer, lower negative emotions, and reduced suicide rates (8–10). Regrettably, studies indicate that the level of meaning of life among cancer patients is not promising, regardless of the stage of diagnosis or treatment (11–13).

Meaning-Centered Group Psychotherapy (MCGP) is a scientifically validated intervention that draws upon Frankl’s work and incorporates existentialism (14, 15). The therapy content blends didactics, discussions, and experiential exercises, focusing on the pivotal aspects of freedom, responsibility, choice, creativity, identity, creative values, experiential values, attitudinal values, and temporality. It aims to enhance the meaning of life of patients diagnosed with advanced cancer. While originally developed for this specific population, MCGP have demonstrated wide applicability, across various populations and cultures, offering relevance and benefits irrespective of the cancer stage or prognosis (14–22).

The meaning of life of cancer patients undergoing active treatment is a critical concern deserving of attention, not only in the late or terminal stages (23). While these patients may not face the same intense physical and emotional challenges as those with advanced cancer, the psychological impact and disruption to their lives should not be underestimated. Taking into account the traditional Chinese culture and the unique characteristics of the population during active treatment, our research team adapted the MCGP and conducted a randomized controlled trial to assess its effectiveness in actively treated cancer patients. The study showed positive effects of MCGP on the meaning of life in aggressively treated cancer individuals (18). However, there are limited studies that have conducted in-depth examinations of the experiences of cancer patients undergoing active treatment with MCGP. Qualitative evidence holds the potential to offer valuable insights into how the MCGP impacts patients’ perspectives and recommendations concerning treatment outcomes. To refine the program before a large-scale randomized controlled trial validating the efficacy of MCGP among Chinese cancer patients, we conducted a qualitative study for invaluable insights.

## 2. Methods

### 2.1. Study design

This study utilized a descriptive qualitative research design, which is rooted in the philosophical principles of naturalistic inquiry. Thematic analysis was employed to describe the expressed, superficial, and apparent experiences of individuals. The research methods and findings were developed and reported in accordance with the Consolidated criteria for reporting qualitative studies (COREQ) (24) (Supplementary File 1).

### 2.2. Participants

Participants for the interview study were selected from a pool of patients who had been randomly assigned to receive MCGP in the RCT study. Inclusion criteria for the RCT study were age between 18 and 60, awareness of cancer diagnosis, Stage I-III cancer, Mandarin fluency, and voluntary participation. Exclusion criteria included acute disease phase, severe hepatic or renal impairment, cardiopulmonary issues, severe mental disorders, significant cognitive deficits, or use of other medications or psychological interventions. Purposive sampling targeted patients with diverse age, education, cancer site, cancer stage, and MCGP session completion. Data collection continued until data saturation. Among the thirty-four MCGP group patients, twenty-two were invited to participate, and twenty-one (95%) agreed, with one declining due to lack of interest.

### 2.3. Intervention

A more comprehensive description of the original MCGP has been published in other articles (15). However, we have made several modifications to the original MCGP, which include: (a) adjusting the intervention frequency to twice a week for 4 weeks; (b) replacing “legacy” with “story” and “achievement” with “satisfaction”; (c) eliminating the experiential exercise in the first session; (d) introducing the experiential exercise “What identities do you want to prioritize after your illness?” in the second activity; (e) incorporating the experiential exercises “What do you consider a meaningful or good death?” and the “Legacy Project”; and (f) placing greater emphasis on “love” and “beauty” in the seventh activity.

### 2.4. Data collection

Patients gave their informed consent prior to the face-to-face interviews conducted in a hospital interview room from June 2022 to September 2022. Interviews were conducted by telephone if the patient had been discharged. Qualitative data was collected using semi-structured interviews conducted within 2 weeks after the completion of MCGP. The research team developed the interview guide based on the study’s purpose and previous work done for MCGP. The initial interview outline was pilot-tested with two subjects and no changes were made to the interview guide, including the pilot interviews in the final analysis (Table 1). The interviews were conducted by a female master’s student in nursing psychology with extensive experience in qualitative research methods. Participants were unfamiliar with the interviewer. Only the researcher and participant were present during each interview, which were recorded and transcribed.

**Table 1:** Interview guide.

What was your experience of the intervention?
What aspects of MCGP did you find most useful? Can you describe some examples of what MCGP helped you with?
What do you think about the sessions (content, format, therapists, homework and environment)?
Do you feel like you have changed after participating in the MCGP?
What challenges were experienced during participation in MCGP?
What are your suggestions on the intervention? What could be improved?
How do you apply the knowledge you have gained in the MCGP in your daily life?

### 2.5. Data analysis

Data analysis occurred concurrently with its collection. No repeat interviews were conducted. The data were managed using NVivo12 (25, 26). The interview data underwent six phases of thematic analysis following Braun and Clarke’s method (27). These phases involved familiarizing with the transcribed data, coding keywords, organizing themes and subthemes, reviewing and naming themes, compiling results, and performing the final analysis. The findings were sent back to the participants to confirm whether the statements accurately reflected their experiences. The analysis was independently conducted by two members of the research team, each possessing prior expertise in qualitative data analysis. Any discrepancies were resolved through discussion and consensus with a third researcher. Peer review was conducted by team members to review the information and coding, ensuring its integrity and minimizing potential research bias.

### 2.6. Ethics

The current study was part of a clinical trial, which obtained ethical approval by the Institutional Review Board of Shanghai Proton and Heavy Ion Hospital (2202-53-04). The study was registered as a clinical trial on Chinese Clinical Trial Registry, number ChiCTR2200060672.

## 3. Results

### 3.1. Sample characteristics

Participants were aged between 22 and 59 years, with a mean of 38 years. Females accounted for 52% of the participants, and the majority (90.5%) had completed college education or beyond. 66.7% of the participants were diagnosed with head and neck cancer (Table 2).

**Table 2:** Participant characteristics (*N* = 21).

Participant	Gender	Age	Education	Tumor site	Cancer stage	Number of MCGP participation
1	Male	25	Post-graduate	Head and Neck	III	8
2	Female	41	College	Breast	I	6
3	Female	59	College	Breast	II	2
4	Female	35	College	Head and Neck	III	7
5	Male	58	HIGH school	Head and Neck	II	8
6	Male	23	College	Head and Neck	I	4
7	Male	44	College	Head and Neck	I	7
8	Male	30	College	Head and Neck	II	7
9	Female	22	College	Head and Neck	I	7
10	Female	37	College	Head and Neck	III	1
11	Female	37	College	Head and Neck	II	8
12	Male	37	College	Head and Neck	I	5
13	Male	33	Post-graduate	Lung cancer	II	8
14	Female	34	College	Head and Neck	II	8
15	Female	47	Post-graduate	Breast	II	8
16	Male	47	High school	Head and Neck	II	7
17	Female	36	Post-graduate	Cervical	II	8
18	Male	37	College	Head and Neck	II	3
19	Female	31	College	Breast	II	7
20	Male	37	College	Head and Neck	II	6
21	Female	41	College	Breast	I	8

### 3.2. Qualitative findings

The interviews lasted an average of 27 min (range 21–43 min). Four main themes and 14 subthemes were identified (Table 3). Illustrative quotes of the themes and subthemes were provided (Supplementary File 2).

**Table 3:** Qualitative themes and subthemes.

Theme	Subtheme
Self-perceived personal change	New insights on things
	Changes in daily lives
	Clear life goals
Overall experience of group therapy	Supportive group environment
	Social Support
	Opinion of Intervention elements
Barriers to participation of MCGP	Evoking emotions
	Physical Limitations
	Lack of Time
	Insufficient acknowledgment of psychosocial care
Suggestions for future interventions	Additional information on conditions
	Rich format and arrangement
	Positive atmosphere
	Homogeneity of the group

### 3.3. Self-perceived personal change

#### 3.3.1. New insights on things

Participants expressed that MCGP delves into broader aspects of life beyond their disease, resulting in many patients gaining a fresh perspective and a deeper understanding of the meaning of life. Through listening to stories shared by fellow patients regarding the power and advantages of family support, some participants underwent a change in their perception of marriage, while others developed a heightened appreciation for the critical role played by family support. Another participant shared that, following her participation in the MCGP, she acquired fresh strategies for cultivating positivity and embracing negative thoughts. Moreover, the themes of the MCGP served as a profound source of inspiration for participants in various ways, particularly the concept of “Attitudinal Sources of Meaning,” which significantly influenced their perceptions of cancer and led them to recognize the inherent value and significance within the experience of the disease.

#### 3.3.2. Changes in daily lives

Numerous participants underwent a profound transformation in their life perspectives, transitioning from states of confusion and apprehension about the future to newfound confidence, hope, and an enhanced sense of personal responsibility. Moreover, they gained a heightened clarity regarding their life priorities. Some expressed a desire to allocate more time and energy to their families, while others stressed the importance of prioritizing their own needs over meeting external expectations. In the bustling lives of many participants, they often felt constrained by limited time for true immersion in life. However, upon delving into “Experiential Sources of Meaning,” patients discovered an enhanced ability to perceive and construct meaning in their everyday experiences.

#### 3.3.3. Clear life goals

Participants gain a clearer perspective on their future goals through their involvement in the MCGP. One participant mentioned that the theme “Historical Sources of Meaning” helped him recognize the importance of legacy and imparting wisdom, providing him with a clearer sense of how he wants to allocate his time and energy in the future. Furthermore, there are patients who, upon realizing their central role in their own lives, muster the courage to pursue long-held desires they had previously set aside.

### 3.4. Overall experience of group therapy

#### 3.4.1. Supportive group environment

Participants underscored the crucial role of the supportive environment cultivated by MCGP, which empowered them to connect with fellow participants and glean valuable insights from diverse viewpoints. They noted that communicating with others who shared similar experiences was often more comfortable than with family members. This safe space provided an outlet for their emotions and an opportunity to address matters related to their illness.

#### 3.4.2. Social support

During interviews, participants highlighted the profound impact of receiving social support from both therapists and peers, noting that this support system can extend beyond their discharge from the hospital.

#### 3.4.3. Opinion of intervention elements

Participants noted that engaging in this type of group activity was a novel experience for them. Surprisingly, they exhibited robust motivation and satisfaction with this format. While most patients found the duration and frequency of the intervention to be suitable and were content with it, a few suggested that the intervention could be slightly shortened. Moreover, many participants expressed positive sentiments regarding the core concepts of the MCGP, firmly believing that these principles held substantial value and warranted further contemplation and discussion.

### 3.5. Barriers to participation of MCGP

#### 3.5.1. Evoking emotions

Discussing illness within a group is nearly inevitable, and this often leads to feelings of sadness and negativity. Consequently, certain participants choose to withdraw from such groups as they find it challenging to cope with these emotionally evocative situations.

#### 3.5.2. Physical limitations

Patients stated that the side effects of the illness and its treatment can make it even harder for them to participate in MCGP. These side effects include problems like reduced taste, oral sores, nausea, vomiting, and others.

#### 3.5.3. Lack of time

Certain participants emphasized the conflicts they faced in balancing their involvement in the MCGP with effectively managing their time, primarily due to work or therapy. These conflicts occasionally rendered them unable to participate in the MCGP.

#### 3.5.4. Insufficient acknowledgment of psychosocial care

Some patients prioritize treating their physical illness when they come to the hospital and overlook the significance of mental health.

### 3.6. Suggestions for future interventions

#### 3.6.1. Additional information on conditions

Participants expressed a desire for additional information on subjects like common side effects after cancer treatment, as well as increased opportunities to communicate with fellow patients about their conditions.

#### 3.6.2. Rich format and arrangement

Participants provided insightful suggestions to enhance the format of experiential exercises and the content of the MCGP, including incorporating positive thinking meditation and presenting the experiential exercises in a gamified format.

#### 3.6.3. Positive atmosphere

Special attention should be given to the fact that some patients have had both positive and negative experiences with the intervention, and these negative experiences may impact their ongoing participation in the MCGP.

#### 3.6.4. Homogeneity of the group

Patients recruited for the study frequently displayed variances in age, disease type, and cancer stage. Some participants mentioned that these variations can lead to a lack of understanding and empathy during discussions about specific topics.

## 4. Discussion

This study aimed to gather insights on the experiences of Chinese cancer patients receiving active treatment while participating in a four-week MCGP, using a qualitative approach.

When confronted with a cancer diagnosis, individuals often grapple with questions about the purpose and meaning of their existence (7, 28). Cultural influences contribute to the tendency for Chinese cancer patients to refrain from openly discussing matters pertaining to their condition with both family members and healthcare professionals (29, 30). The MCGP offers a supportive space wherein patients can openly explore their life experiences, emotions, relationships, and the profound impact the disease has had on them. Simultaneously, exploring the core concepts of the MCGP seemed to furnish patients with a structured approach for tackling these profound existential inquiries, bestowing upon them invaluable insights into the inherent worth of life itself. This process fosters a deeper level of introspection among patients, resulting in a fresh perspective, a clarified life purpose, and a sense of meaning by prioritizing what truly matters to them. These findings are further corroborated by the results of qualitative studies involving the application of MCGP to Portuguese cancer patients and Spanish patients dealing with advanced cancer (22, 31). Most patients not only appreciated the overall value of the MCGP but also found significant benefit in the individual themes (32, 33). Different themes held varying degrees of value and meaning for different patients. Moreover, our results illustrate that some patients successfully applied the insights gained from the MCGP to their daily lives post-intervention. This could potentially explain why certain trials have reported enduring effects of the MCGP (14, 15, 17, 19). However, further research is required to validate this suppose.

Patients frequently highlight their appreciation for gaining fresh perspectives through interactions with therapists or peers, a phenomenon that finds resonance in the MCGP research applied to both cancer survivors and Spanish patients with advanced cancer (22, 34). This aptly underscores the distinctive strengths inherent in the group intervention format. Nearly all patients in our group provided positive feedback on this intervention format, aligning with earlier findings that suggest Chinese immigrant cancer patients favor the group format when engaging in MCP (35). Additionally, patients found the exploration of the core concepts of the MCGP to be meaningful, aligning with the feedback received from cancer survivors and Latino advanced cancer patients regarding the concept of MCP (34, 36). This implies that addressing existential issues is imperative, irrespective of a cancer patient’s stage of treatment—whether it be during active treatment, in the advanced stage, or post-treatment—and regardless of their cultural background. Before participating in the intervention, patients may not have previously considered such profound existential questions. The MCGP served as a vital catalyst, enabling deep self-reflection, enhancing their capacity to find meaning in their lives and experiences, and offering patients multiple avenues to gain a sense of meaning.

Some patients discontinued their involvement due to the heavy atmosphere in the group, leading to feelings of distress. This observation aligns with the findings of the application of MCP for palliative care patients, highlighting the persistent challenge of delving into the existential topic, both during active treatment and at the end of life (33). The expression of emotions in the group had two sides: some patients saw it as an opportunity to express and release emotions, while others chose not to participate due to the somber atmosphere. A few patients initially struggled in the first two sessions but experienced relief and relaxation from the third session onwards. This underscores the significance of acknowledging and addressing negative emotions during the early stages of our group discussions. Furthermore, in contrast to the application of MCGP in patients with advanced cancer, where missed visits were primarily due to severe illness (37), patients undergoing active cancer treatment faced challenges related to both physical discomfort from treatment and time constraints due to work commitments. This emphasizes the need for flexibility in the format of the MCGP. Future studies can overcome these obstacles by using suitable formats that align with patient preferences and needs, including online platforms. The online implementation of the MCP has shown positive outcomes both among parents who have experienced the loss of a child to cancer and cancer caregivers (38, 39). In addition, patients often underestimate the importance of psychosocial care compared to disease treatment. Therefore, it is crucial to raise patients’ awareness about the critical role of psychosocial well-being in overall health.

Based on participants’ feedback, we can improve the follow-up study by including disease-related health information to alleviate patients’ anxiety and uncertainty about their condition (40, 41). Visual aids and games can be effective tools to enrich the experiential exercises and enhance participants’ self-expression. Additionally, future studies could refine the age range and assess the specific type and stage of patients’ diseases, considering the significant influence of age on one’s perception of life’s meaning. Creating more homogeneous groups based on these factors would likely foster a stronger sense of empathy and connection among participants.

### 4.1. Limitations

In line with the prevalent composition of cancer patients at our hospital, over half of the participants in this study were head and neck cancer patients. It is imperative to exercise caution when extrapolating our findings to other diagnoses or more diverse patient populations. Also, the average age of our participants was 38 years, which is younger than in other MCP studies (15, 19). This means that our findings may not directly apply to other age groups within the cancer population. Additionally, we were unable to include the perspectives of participants who did not attend the MCGP in our interviews. As a result, we could not explore their reasons for not participating in the MCGP or understand the barriers and motivations that influenced their decision-making process.

### 4.2. Clinical implications

This study sheds light on the experiences of Chinese cancer patients undergoing active treatment who participated in MCGP. While most MCGP studies have focused on Western cultures, our study specifically examines the Chinese context. We have identified key factors influencing patient adherence and program participation, providing insights for the development of tailored psychosocial interventions for Chinese cancer patients. It is noteworthy that meaning-centered therapy is recommended as a psychotherapeutic approach in Chinese cancer psychotherapy guidelines for patients with progressive cancer. Based on our findings, we propose integrating this intervention program into the existing supportive care during the active treatment phase to enhance patients’ psychological well-being.

## 5. Conclusion

This study offers valuable insights into the effectiveness of MCGP for cancer patients undergoing active treatment, along with highlighting the challenges encountered and providing recommendations for improving engagement. Future research endeavors could focus on adapting the MCGP to better align with cultural and study population characteristics, while also assessing its effectiveness across various age groups, cancer types, and stages of cancer.

## Data availability statement

The data analyzed in this study is subject to the following licenses/restrictions: The data are not publicly available due to privacy or ethical restrictions. Available upon request. Requests to access these datasets should be directed to SW, 22111170004@m.fudan.edu.cn.

## Ethics statement

The studies involving humans were approved by Shanghai Proton and Heavy Ion Center. The studies were conducted in accordance with the local legislation and institutional requirements. The participants provided their written informed consent to participate in this study. Written informed consent was obtained from the individual(s), and minor(s)' legal guardian/next of kin, for the publication of any potentially identifiable images or data included in this article.

## Author contributions

SW: Conceptualization, Formal analysis, Methodology, Project administration, Writing – original draft. MZ: Formal analysis, Methodology, Project administration, Software, Writing – original draft. YZ: Formal analysis, Methodology, Writing – review & editing. LZ: Project administration, Resources, Writing – review & editing. XL: Methodology, Project administration, Writing – review & editing. HW: Project administration, Supervision, Writing – review & editing, Funding acquisition.

## References

[1] Sung H Ferlay J Siegel RL Laversanne M Soerjomataram I Jemal A. Global Cancer statistics 2020: Globocan estimates of incidence and mortality worldwide for 36 cancers in 185 countries. CA Cancer J Clin. (2021) 71:209–49. doi: 10.3322/caac.21660 33538338

[2] Rhoten BA Murphy BA Dietrich MS Ridner SH. Depressive symptoms, social anxiety, and perceived neck function in patients with head and neck Cancer. Head Neck. (2018) 40:1443–52. doi: 10.1002/hed.25129, PMID: 29573016 PMC6037559

[3] Herschbach P Britzelmeir I Dinkel A Giesler JM Herkommer K Nest A. Distress in Cancer patients: who are the Main groups at risk? Psycho-Oncology. (2020) 29:703. doi: 10.1002/pon.5321 31876011

[4] LeMay K Wilson KG. Treatment of existential distress in life threatening illness: a review of manualized interventions. Clin Psychol Rev. (2008) 28:472–93. doi: 10.1016/j.cpr.2007.07.013, PMID: 17804130

[5] Yong J Kim J Han SS Puchalski CM. Development and validation of a scale assessing spiritual needs for Korean patients with Cancer. J Palliat Care. (2008) 24:240–6. doi: 10.1177/082585970802400403, PMID: 19227015

[6] Hsiao SM Gau ML Ingleton C Ryan T Shih FJ. An exploration of spiritual needs of Taiwanese patients with advanced Cancer during the therapeutic processes. J Clin Nurs. (2011) 20:950–9. doi: 10.1111/j.1365-2702.20 21044187

[7] Carreno DF Eisenbeck N. Existential insights in Cancer: Meaning in life adaptability. Medicina. (2022) 58:58. doi: 10.3390/medicina58040461, PMID: 35454300 PMC9029503

[8] Testoni I Sansonetto G Ronconi L Rodelli M Baracco G Grassi L. Meaning of life, representation of death, and their association with psychological distress. Palliat Support Care. (2018) 16:511–9. doi: 10.1017/s1478951517000669 28789719

[9] Lee V Cohen SR Edgar L Laizner AM Gagnon AJ. Meaning-making and psychological adjustment to Cancer: development of an intervention and pilot results. Oncol Nurs Forum. (2006) 33:291–302. doi: 10.1188/06.Onf.291-302, PMID: 16518445

[10] Breitbart W Rosenfeld B Pessin H Kaim M Funesti-Esch J Galietta M. Depression, hopelessness, and desire for hastened death in terminally ill patients with Cancer. JAMA. (2000) 284:2907-11. doi: 10.1001/jama.284.22.2907, PMID: 11147988

[11] Sleight AG Boyd P Klein WMP Jensen RE. Spiritual peace and life meaning may buffer the effect of anxiety on physical well-being in newly diagnosed Cancer survivors. Psychooncology. (2021) 30:52–8. doi: 10.1002/pon.5533, PMID: 32840948

[12] Scrignaro M Bianchi E Brunelli C Miccinesi G Ripamonti CI Magrin ME. Seeking and experiencing meaning: exploring the role of meaning in promoting mental adjustment and Eudaimonic well-being in Cancer patients. Palliat Support Care. (2015) 13:673–81. doi: 10.1017/s1478951514000406, PMID: 24825576

[13] Liu X Liu Z Cheng Q Xu N Liu H Ying W. Effects of meaning in life and individual characteristics on dignity in patients with advanced Cancer in China: a cross-sectional study. Support Care Cancer. (2021) 29:2319–26. doi: 10.1007/s00520-020-05732-2, PMID: 32914328

[14] Breitbart W Rosenfeld B Gibson C Pessin H Poppito S Nelson C. Meaning-centered group psychotherapy for patients with advanced Cancer: a pilot randomized controlled trial. Psycho-Oncology. (2010) 19:21–8. doi: 10.1002/pon.1556, PMID: 19274623 PMC3648880

[15] Breitbart W Rosenfeld B Pessin H Applebaum A Kulikowski J Lichtenthal WG. Meaning-centered group psychotherapy: an effective intervention for improving psychological well-being in patients with advanced Cancer. J Clin Oncol. (2015) 33:749–54. doi: 10.1200/jco.2014.57.2198, PMID: 25646186 PMC4334778

[16] Goldezweig G Hasson-Ohayon I Elinger G Laronne A Wertheim R Pizem N. Adaptation of meaning centered group psychotherapy (Mcgp) in the Israeli context: the process of importing an intervention and preliminary results In: Breitbart W, editor. Meaning centered psychotherapy in the Cancer setting: Finding meaning and Hope in the face of suffering. New York: Oxford University Press (2016). 145–56.

[17] van der Spek N Vos J van Uden-Kraan CF Breitbart W Cuijpers P Holtmaat K. Efficacy of meaning-centered group psychotherapy for Cancer survivors: a randomized controlled trial. Psychol Med. (2017) 47:1990–2001. doi: 10.1017/s0033291717000447, PMID: 28374663 PMC5501751

[18] Wang S Zhu Y Wang Z Zheng M Li X Zhang Y. Efficacy of meaning-centered group psychotherapy in Chinese patients with Cancer: a randomized controlled trial. Palliat Support Care. (2023):1–9. doi: 10.1017/s1478951523000998, PMID: 37558651

[19] Holtmaat K van der Spek N Lissenberg-Witte B Breitbart W Cuijpers P Verdonck-de LI. Long-term efficacy of meaning-centered group psychotherapy for Cancer survivors: 2-year follow-up results of a randomized controlled trial. Psychooncology. (2020) 29:711–8. doi: 10.1002/pon.5323, PMID: 31876012 PMC7199891

[20] da Ponte G Ouakinin S Santo JE Ohunakin A Prata D Amorim I. Meaning-centered group psychotherapy in Portuguese Cancer patients: a pilot exploratory trial. Palliat Support Care. (2021) 19:464–73. doi: 10.1017/s1478951521000602, PMID: 34039464 PMC8324537

[21] Applebaum AJ Kulikowski JR Breitbart W. Meaning-centered psychotherapy for Cancer caregivers (Mcp-C): rationale and overview. Palliat Support Care. (2015) 13:1631–41. doi: 10.1017/s1478951515000450, PMID: 26000705 PMC5084443

[22] Fraguell C Limonero JT Gil F. Psychological aspects of meaning-centered group psychotherapy: Spanish experience. Palliat Support Care. (2018) 16:317–24. doi: 10.1017/s1478951517000293, PMID: 29877783

[23] Lee V Loiselle CG. The salience of existential concerns across the Cancer control continuum. Palliat Support Care. (2012) 10:123–33. doi: 10.1017/s1478951511000745, PMID: 22464280

[24] Tong A Sainsbury P Craig J. Consolidated criteria for reporting qualitative research (Coreq): a 32-item checklist for interviews and focus groups. Int J Qual Health Care. (2007) 19:349–57. doi: 10.1093/intqhc/mzm042, PMID: 17872937

[25] Thomas J Harden A. Methods for the thematic synthesis of qualitative research in systematic reviews. BMC Med Res Methodol. (2008) 8:45. doi: 10.1186/1471-2288-8-45, PMID: 18616818 PMC2478656

[26] Houghton C Murphy K Meehan B Thomas J Brooker D Casey D. From screening to synthesis: using Nvivo to enhance transparency in qualitative evidence synthesis. J Clin Nurs. (2017) 26:873–81. doi: 10.1111/jocn.13443 27324875

[27] Braun V Clarke V. Using thematic analysis in psychology. Qual Res Psychol. (2006) 3:77–101. doi: 10.1191/1478088706qp063oa

[28] Park CL Edmondson D Fenster JR Blank TO. Meaning making and psychological adjustment following Cancer: the mediating roles of growth, life meaning, and restored just-world beliefs. J Consult Clin Psychol. (2008) 76:863–75. doi: 10.1037/a0013348, PMID: 18837603

[29] Zheng RS Guo QH Dong FQ Owens RG. Chinese oncology Nurses' experience on caring for dying patients who are on their final days: a qualitative study. Int J Nurs Stud. (2015) 52:288–96. doi: 10.1016/j.ijnurstu.2014.09.009, PMID: 25445033

[30] Chen C Cheng G Chen X Yu L. Information disclosure to Cancer patients in mainland China: a Meta-analysis. Psychooncology. (2023) 32:342–55. doi: 10.1002/pon.6085 36582008

[31] da Ponte G Ouakinin S Santo JE Amorim I Gameiro Z Fitz-Henley M. Process of therapeutic changes in meaning-centered group psychotherapy adapted to the Portuguese language: a narrative analysis. Palliat Support Care. (2020) 18:254–62. doi: 10.1017/s147895151900110x, PMID: 31957635 PMC7332394

[32] Applebaum AJ Roberts KE Lynch K Gebert R Loschiavo M Behrens M. A qualitative exploration of the feasibility and acceptability of meaning-centered psychotherapy for Cancer caregivers. Palliat Support Care. (2022) 20:623–9. doi: 10.1017/s1478951521002030, PMID: 35078552 PMC9314455

[33] Rosenfeld B Saracino R Tobias K Masterson M Pessin H Applebaum A. Adapting meaning-centered psychotherapy for the palliative care setting: results of a pilot study. Palliat Med. (2017) 31:140–6. doi: 10.1177/0269216316651570, PMID: 27435603 PMC5473503

[34] van der Spek N van Uden-Kraan CF Vos J Breitbart W Tollenaar RA van Asperen CJ. Meaning-centered group psychotherapy in Cancer survivors: a feasibility study. Psychooncology. (2014) 23:827–31. doi: 10.1002/pon.3497, PMID: 24991747

[35] Leng J Lui F Huang X Breitbart W Gany F. Patient perspectives on adapting meaning-centered psychotherapy in advanced Cancer for the Chinese immigrant population. Support Care Cancer. (2019) 27:3431–8. doi: 10.1007/s00520-019-4638-2, PMID: 30661201 PMC6642030

[36] Torres-Blasco N Rosario-Ramos L Navedo ME Peña-Vargas C Costas-Muñiz R Castro-Figueroa E. Importance of communication skills training and meaning centered psychotherapy concepts among patients and caregivers coping with advanced Cancer. Int J Environ Res Public Health. (2023) 20:20. doi: 10.3390/ijerph20054458, PMID: 36901468 PMC10002270

[37] Applebaum AJ Lichtenthal WG Pessin HA Radomski JN Simay Gökbayrak N Katz AM. Factors associated with attrition from a randomized controlled trial of meaning-centered group psychotherapy for patients with advanced Cancer. Psychooncology. (2012) 21:1195–204. doi: 10.1002/pon.2013, PMID: 21751295 PMC3827859

[38] Lichtenthal WG Catarozoli C Masterson M Slivjak E Schofield E Roberts KE. An open trial of meaning-centered grief therapy: rationale and preliminary evaluation. Palliat Support Care. (2019) 17:2–12. doi: 10.1017/s1478951518000925, PMID: 30683164 PMC6401220

[39] Applebaum AJ Buda KL Schofield E Farberov M Teitelbaum ND Evans K. Exploring the Cancer Caregiver's journey through web-based meaning-centered psychotherapy. Psychooncology. (2018) 27:847–56. doi: 10.1002/pon.4583, PMID: 29136682 PMC8045418

[40] Guccione L Fisher K Mileshkin L Tothill R Bowtell D Quinn S. Uncertainty and the unmet informational needs of patients with Cancer of unknown primary (cup): a cross-sectional multi-site study. Support Care Cancer. (2022) 30:8217–29. doi: 10.1007/s00520-022-07228-7, PMID: 35804177 PMC9512714

[41] Venetis MK Staples S Robinson JD Kearney T. Provider information provision and breast Cancer patient well-being. Health Commun. (2019) 34:1032–42. doi: 10.1080/10410236.2018.1454254 29583022

