# Supplementary material for: Exploring the experience of meaning-centered group psychotherapy among Chinese cancer patients during active treatment: a descriptive qualitative study
Source: Front Psychiatry. 2023 Oct 6;14:1264257. doi: 10.3389/fpsyt.2023.1264257 (PMC10590054; doi:10.3389/fpsyt.2023.1264257)
Supplement: Supplementary file 1 [file Table_1.docx]

| **Theme/Subthemes** | **Supporting quotes** |
| --- | --- |
| **Self-perceived personal change** | |
| New insights on things | - Life is actually not in the length. I may now be more inclined or more enthusiastic to increase its breadth and thickness[P12]. - The experience of this disease has brought clarity to my previously uncertain life. In fact, it is still from the disadvantage to find some of the most beneficial value for you[P20]. - I used to prioritize different aspects of marriage, but now I'm realizing that mutual support and assistance during times of hardship are more crucial than receiving help in other areas from your partner[P12]. - I think we are most fortunate that the couple is in harmony, we are all very lucky that we have a family to support us, so we have to cheer even more[P17]. - Initially, I sought to eliminate negative energy from my body, Now I realized my perspective was flawed. Through absorbing meaningful experiences, my negative energy eventually vanished[P14]. |
| Changes in daily lives | - After this month, I became more open to facing it and started actively seeking a better treatment plan. Now, I have more hope for the future and a renewed sense of purpose in living[P1]. - I didn't like flowers, but after joining this group, I wanted to try smelling them, I felt so comfortable and thought of a certain moment when I was a child, I was very impressed[P19]. - I owe a lot to my daughter, my wife and my whole family. I want to be able to take up my role as a responsible person after I have adjusted as soon as possible after the next treatment[P12]. - I'm actually not worried about my son at all right now, and I think even without me, he can still grow up very well and healthy, so I still want to put more energy into myself in the future[P17]. - A nurse advised me to incorporate more walking into my routine. Although I used to dislike walking, I have now made it a daily habit. I believe it is beneficial for my body, and this change in my mindset has been quite significant[P20]. |
| Clear life goals | - I now realize that I spent my life chasing temporary rewards, and I have a newfound desire to pursue something more meaningful for my family and others who share similar experiences[P2]. - I've been wanting to travel for a long time, but I'm always worried about the kids... I'm definitely going to do it when I get out of the hospital this time... [P4]. |
| **Overall experience of group therapy** | |
| Supportive group environment | - In these group activities, on the one hand there is an exchange with each other and on the other hand there is an increase in wisdom[P21]. - I learned various perspectives from the group members, which may shine from time to time in my future life with cancer survival in the long run, and then give me some inspiration and strength[P11]. - Talking to you in a group will be more relaxed than when I talk to my family. I feel that they understand me better[P15]. - Talking with them, I also learned some knowledge about cancer that I didn't know before, and now I think it's not that scary[P5]. |
| Social Support | - My ward was next to both of them, and because we both had nasopharyngeal cancer and had no sense of taste when we ate, we ate together and encouraged each other to cheer up[P8]. - I was warmed by xxx's response to me in the group, and I think we'll be in touch after we're discharged[P8]. - Sometimes the feedback from those two teachers may enlighten me and make my ideas feel a little clearer, they are more clearly expressed and the ideas are a little clearer[P13]. |
| Opinion of intervention elements | - It's great that this month-long event coincides with our hospital stay and adds a lot of fun to our boring hospital life[P2]. - We are usually quite bored, radiotherapy is only half an hour a day, there is a lot of free time, this time is quite suitable[P6]. - Today, I attended a lecture where someone presented using PowerPoint slides from above while we listened from below. However, I prefer our format, which enables us to gain a deeper understanding of each person's experiences and fully express their thoughts[P16]. - I have a bad back, sometimes I sit for two hours at a time, it's a bit unbearable, it would be nice to shorten the time a little bit[P2]. - If I hadn't joined this group, I likely wouldn't have considered certain things. However, the topics we discuss in this group are extremely important to each one of us[P9]. |
| **Barriers to participation of MCGP** | |
| Evoking emotions | - I had already adjusted myself, so I was less inclined to accept some of the negativity from others[P10]. - xxx is a recurrent cancer patient and it was hard to hear his story. This is the first time I had cancer and I didn't realize it would come back[P18]. |
| Physical Limitations | - Because I have head and neck cancer, I will have severe oral mucositis in the late stage of treatment, and I don't want to talk at this time because it hurts[P4]. - If I had chemotherapy on that day, I would feel sick and throw up throughout the day, making me want to stay in bed[P18]. |
| Lack of Time | - I'm here in the hospital but my work hasn't stopped, sometimes I need to make work calls or have work meetings, which may conflict with our activities[P7]. - Sometimes just in time for treatment, there is really no way to come. We definitely still focus on treatment[P3]. |
| Insufficient acknowledgment of psychosocial care | - I'm here to heal, and I think it's a waste of time to participate in this. Will it make me well? [P10] |
| **Suggestions for future interventions** | |
| Additional information on conditions | - This event is more about thinking about life, but we also need to know more about the disease, I hope to add this piece later, I believe we will all need[P5]. - It's great that we're discussing these topics, but it would be nice if there were dedicated opportunities for those of us who are sick to communicate about our conditions[P8]. |
| Rich format and arrangement | - These exercises are more demanding on our expressive skills, can we add some other forms of activities, such as meditation. I have heard before that this is quite useful[P15]. - I've done a "lifeline" game before, and I thought it might be more appealing to us if we could express some of the exercises in the form of some images or games[P21]. |
| Positive atmosphere | - This group was a new experience for me. Actually, the first two experiences were not very good because the atmosphere was a bit sad, but I am very glad that I persevered. I know there are some patients who did not continue to participate because of this reason[P14]. |
| Homogeneity of the group | - I'm the oldest in the group, and sometimes I'm afraid they won't understand if I talk too much about my grandchildren[P3]. - Because my nasopharyngeal cancer was detected relatively early and I did not need chemotherapy, I sometimes dare not say I am quite lucky. I know some of them are quite serious[P9]. |
